# Sexual Minorities in England Have Poorer Health and Worse Health Care Experiences: A National Survey

**DOI:** 10.1007/s11606-014-2905-y

**Published:** 2014-09-05

**Authors:** Marc N. Elliott, David E. Kanouse, Q Burkhart, Gary A. Abel, Georgios Lyratzopoulos, Megan K. Beckett, Mark A. Schuster, Martin Roland

**Affiliations:** 1 RAND Corporation, 1776 Main Street, Santa Monica, CA 90401-3208 USA; 2 Institute of Public Health, Cambridge Centre for Health Services Research University of Cambridge, Robinson Way, Cambridge, CB2 0SR UK; 3 Boston Children’s Hospital/Harvard Medical School, Boston, Massachusetts, USA

**Keywords:** sexual orientation, health care experiences, disparities

## Abstract

**BACKGROUND:**

The health and healthcare of sexual minorities have recently been identified as priorities for health research and policy.

**OBJECTIVE:**

To compare the health and healthcare experiences of sexual minorities with heterosexual people of the same gender, adjusting for age, race/ethnicity, and socioeconomic status.

**DESIGN:**

Multivariate analyses of observational data from the 2009/2010 English General Practice Patient Survey.

**PARTICIPANTS:**

The survey was mailed to 5.56 million randomly sampled adults registered with a National Health Service general practice (representing 99 % of England’s adult population). In all, 2,169,718 people responded (39 % response rate), including 27,497 people who described themselves as gay, lesbian, or bisexual.

**MAIN MEASURES:**

Two measures of health status (fair/poor overall self-rated health and self-reported presence of a longstanding psychological condition) and four measures of poor patient experiences (no trust or confidence in the doctor, poor/very poor doctor communication, poor/very poor nurse communication, fairly/very dissatisfied with care overall).

**KEY RESULTS:**

Sexual minorities were two to three times more likely to report having a longstanding psychological or emotional problem than heterosexual counterparts (age-adjusted for 5.2 % heterosexual, 10.9 % gay, 15.0 % bisexual for men; 6.0 % heterosexual, 12.3 % lesbian and 18.8 % bisexual for women; *p* < 0.001 for each). Sexual minorities were also more likely to report fair/poor health (adjusted 19.6 % heterosexual, 21.8 % gay, 26.4 % bisexual for men; 20.5 % heterosexual, 24.9 % lesbian and 31.6 % bisexual for women; *p* < 0.001 for each).

Adjusted for sociodemographic characteristics and health status, sexual minorities were about one and one-half times more likely than heterosexual people to report unfavorable experiences with each of four aspects of primary care. Little of the overall disparity reflected concentration of sexual minorities in low-performing practices.

**CONCLUSIONS:**

Sexual minorities suffer both poorer health and worse healthcare experiences. Efforts should be made to recognize the needs and improve the experiences of sexual minorities. Examining patient experience disparities by sexual orientation can inform such efforts.

**Electronic supplementary material:**

The online version of this article (doi:10.1007/s11606-014-2905-y) contains supplementary material, which is available to authorized users.

## INTRODUCTION

The health and healthcare of sexual minorities, including gay, lesbian, bisexual, and transgender people, have recently been identified as priority areas for research.^1^,^2^ Because of the paucity of research using large, representative samples, much of what is known about the health of sexual minorities comes from small samples that may not accurately represent national populations. Relatedly, studies have tended to combine sexual minority groups that may be quite different in their health and experiences with health care.

Despite these limitations, past research suggests that the physical and mental health of sexual minority populations differs in several ways from that of the general population.^3^ For example, compared with heterosexual people, gay and lesbian people have higher prevalence of mental health problems, including depression, anxiety and substance abuse.^4^–^7^ A California study found that gay men and lesbian and bisexual women reported more health conditions and limitations than heterosexuals; differences for women disappeared when controlling for psychological distress.^8^ A Massachusetts study found that sexual minorities were less likely than heterosexual adults to report “excellent” overall health.^9^ However, a study of US adult male couples did not replicate these findings,^10^ consistent with a broader literature finding better health for partnered than single adults.

Discrimination may affect the quality of care that sexual minorities receive.^2^ Some healthcare workers may be uncomfortable communicating with sexual minority patients and insensitive to their needs.^1^,^2^,^11^–^13^ Sexual minorities may be reluctant to disclose their orientation to doctors whom they view as unsympathetic.^14^–^17^ Consistent with such concerns, US sexual minorities report negative experiences when disclosing their sexual orientation, including denial of care, verbal abuse, and disrespectful behavior.^18^–^20^

The English General Practice Patient Survey (GPPS) is a large national health survey of health and healthcare experiences that includes a sexual orientation question.^21^,^22^ It provides a unique opportunity to study the associations among sexual orientation, sociodemographic characteristics, and health in a large population-based sample. We contrast the health and healthcare experiences of sexual minorities (here limited to gay/lesbian and bisexual men and women) with those of heterosexual men and women.

## METHODS

We used data from the 2009/10 GPPS, a nationally administered survey of patient experience with primary care.^23^ In the UK, 99 % of the resident population is registered with a general practice and services are largely free at the point of use. Between April 2009 and March 2010, the survey was mailed to 5.56 million randomly selected patients registered with general practices. Because these data contain no patient identifiers, the institutional review board at the Cambridge Centre for Health Services Research found the study to be exempt from review.

Patients were asked about their health, healthcare experiences, and personal characteristics (race/ethnicity, religion, and sexual orientation). The question about sexual orientation is also used in UK Office of National Statistics Social Surveys^24^: “Which of the following best describes how you think of yourself? Heterosexual/straight; Gay/lesbian; Bisexual; Other; I would prefer not to say.”

Self-identified race/ethnicity was recorded in six groups (White, Mixed, South Asian, Black, Chinese and Other) using the Office for National Statistics’ classification scheme.^25^ Socioeconomic characteristics based on the patient’s postcode were measured using quintiles of the Index of Multiple Deprivation. This index synthesizes community-level income, employment, education, living environment, crime, and other factors.^26^ An indicator of each patient’s general practice was coded to distinguish within- and between-practice differences in patient experience. General health status was measured with a single question: “In general, would you say your health is excellent, very good, good, fair, or poor?”^27^,^28^ Responses were dichotomized into fair/poor versus good/very good/excellent, a common classification. Finally, respondents were asked an item from the UK’s 2011 Census,^29^ “Do you have any of the following long-standing conditions? Please include problems which are due to old age.” They were then presented with a list of six long-term health problems, which included “a long-standing psychological or emotional condition.”

We used four dichotomous measures of poor patient experience from the GPPS regarding experiences with physicians and nurses: 1) “no” trust or confidence in the doctor; 2) having at least one “poor” or “very poor” response to the seven doctor communication questions (giving enough time, asking about symptoms, listening, explaining tests and treatments, involving in decisions, treating with care and concern, and taking problems seriously); 3) having at least one “poor” or “very poor” response to at least one of seven nurse communication questions (similar to those regarding the doctor); and 4) being “fairly” or “very” dissatisfied with care overall (See Appendix Table S1 [available online] for complete patient experience questions).

To determine whether the general or psychological health of sexual minority patients differed from that of heterosexual/straight patients of the same gender, we employed multivariable logistic regression models controlling for age, race/ethnicity, and deprivation quintile. The dependent variables were (a) reporting fair/poor overall health, and (b) reporting the presence of a longstanding psychological condition.

All analyses were stratified by gender and used all available cases for each analysis. We used weights to improve representativeness of respondents in terms of age, gender and practice.^30^ Analyses were executed in SAS 9.2 (SAS Institute Inc., Cary, NC, USA). To analyze the association between sexual orientation and healthcare experiences, we estimated a series of separate logistic regression models for men and women for each of the four patient experience measures. Model 1 (fixed effects only) estimates overall differences in patient experiences associated with sexual orientation, adjusting for patient age, race/ethnicity, deprivation, and five-category general health status, but not adjusting for practice. Model 2 adds random practice effects, so that only within-practice differences in patient experience are estimated. By comparing the results to those from Model 1, we can assess how much of any difference by sexual orientation is due to the concentration of sexual minority patients in lower-performing practices. Lastly, in a supplementary analysis we explored whether the degree of disparities differed across general practices using linear mixed models that added lesbian, gay, and bisexual by practice random effects to Model 2.

All results from logistic regression analysis are presented as covariate-adjusted proportions using predictive margins.^31^ We present the resulting values so that we can describe the proportion of a given sexual orientation and gender group (e.g., lesbians) predicted to experience a given outcome (e.g., poor or fair health) if each group had the same distribution of covariates (e.g., age, race/ethnicity, and socioeconomic deprivation) as seen in the entire population, thereby quantifying the population-level impact of differences associated with sexual orientation.

## RESULTS

There were 2,169,718 survey respondents (a 39 % response rate). Weighted analyses showed that 86.8 % of men reported being heterosexual/straight, 1.7 % gay, 0.5 % bisexual, 0.6 % “other,” 4.4 % preferred not to say, and 6.0 % did not answer the question. Among women, 86.1 % reported being heterosexual/straight, 0.6 % lesbian, 0.5 % bisexual, 0.6 % “other”, 4.0 % preferred not to say, and 8.3 % did not answer the question.

The characteristics of those who endorsed heterosexual/straight compared with those who responded gay, lesbian, bisexual, “other”, and those who declined to say or gave no response appear in Table 1. Men and women who described themselves as gay/lesbian or bisexual were substantially younger than the rest of the population. Older patients were more likely than younger patients both to record their orientation as “other” and to leave the question unanswered. There were also marked differences by race/ethnicity: for both men and women, there were proportionately fewer Asians and Blacks among gay and lesbian patients and proportionately more Asians and Blacks among bisexual and “other” patients. Sexual minority patients were more likely to live in deprived areas: 19 % of heterosexual men lived in areas in the most deprived quintile, compared with 29 % of gay men and 31 % of bisexual men. Similar differences were found among women. Additional sensitivity analyses not reported here found that all differences in deprivation persisted within gender (*p* < 0.001) after controlling for age and race/ethnicity.

**Table 1:** Demographic Characteristics of Patients by Sexual Orientation (Weighted Percentages)

	**Percent Distribution by Response Category (Men**)				
	**Heterosexual** (**Ref**.)	**Gay**	**Bisexual**	**Other**	Prefer Not to Say/Missing*
	*n* = 764,291	*n* = 12,346	*n* = 4,161	*n* = 6,167	*n* = 110,361
**Age**					
18–24 (*n* = 103,432)	10.3	**12.6** ***p*** **< 0.001**	**14.2** ***p*** **< 0.001**	10.1 *p* = 0.82	10.2 *p* = 0.73
25–34 (*n* = 229,680)	16.8	**24.5** ***p*** **< 0.001**	**22.0** ***p*** **< 0.001**	**19.1** ***p*** **= 0.001**	**16.2** ***p*** **= 0.007**
35–44 (*n* = 325,195)	19.8	**29.2** ***p*** **< 0.001**	20.1 *p* = 0.74	21.1 *p* = 0.09	**18.6** ***p*** **< 0.001**
45–54 (*n* = 374,901)	18.8	**20.0** ***p*** **= 0.003**	18.5 *p* = 0.68	**16.8** ***p*** **= 0.005**	**16.0** ***p*** **< 0.001**
55–64 (*n* = 427,004)	16.1	**8.8** ***p*** **< 0.001**	**13.0** ***p*** **< 0.001**	**11.2** ***p*** **< 0.001**	**13.4** ***p*** **< 0.001**
65–74 (*n* = 354,733)	10.8	**3.4** ***p*** **< 0.001**	**7.3** ***p*** **< 0.001**	10.2 *p* = 0.16	**12.4** ***p*** **< 0.001**
75–84 (*n* = 221,209)	5.9	**1.2** ***p*** **< 0.001**	**3.7** ***p*** **< 0.001**	**8.1** ***p*** **< 0.001**	**9.7** ***p*** **< 0.001**
85+ (*n* = 65,407)	1.6	**0.3** ***p*** **< 0.001**	1.1 *p* = 0.02	**3.2** ***p*** **< 0.001**	**3.5** ***p*** **< 0.001**
**Race/ethnicity**					
White (*n* = 1,821,689)	89.5	**92.0** ***p*** **< 0.001**	**75.0** ***p*** **< 0.001**	**62.4** ***p*** **< 0.001**	**68.2** ***p*** **< 0.001**
Mixed (*n* = 15,872)	0.8	**1.5** ***p*** **< 0.001**	**1.5** ***p*** **< 0.001**	**1.9** ***p*** **< 0.001**	**1.4** ***p*** **< 0.001**
Asian (*n* = 110,904)	4.5	**1.5** ***p*** **< 0.001**	**11.7** ***p*** **< 0.001**	**14.0** ***p*** **< 0.001**	**15.7** ***p*** **< 0.001**
Black (*n* = 56,901)	2.1	**0.9** ***p*** **< 0.001**	**5.2** ***p*** **< 0.001**	**7.5** ***p*** **< 0.001**	**4.9** ***p*** **< 0.001**
Chinese (*n* = 9,859)	0.5	**0.8** ***p*** **< 0.001**	0.6 *p* = 0.48	**1.6** ***p*** **< 0.001**	**1.1** ***p*** **< 0.001**
Other (*n* = 67,255)	2.6	**3.4** ***p*** **< 0.001**	**6.1** ***p*** **< 0.001**	**12.7** ***p*** **< 0.001**	**8.7** ***p*** **< 0.001**
**Deprivation Quintiles (IMD)**					
1 = Least Deprived (*n* = 366,364)	21.0	**11.6** ***p*** **< 0.001**	**12.1** ***p*** **< 0.001**	**9.4** ***p*** **< 0.001**	**12.9** ***p*** **< 0.001**
2 (*n* = 402,019)	20.6	**14.0** ***p*** **< 0.001**	**15.5** ***p*** **< 0.001**	**11.6** ***p*** **< 0.001**	**14.4** ***p*** **< 0.001**
3 (*n* = 423,774)	20.1	19.0 *p* = 0.03	**17.5** ***p*** **= 0.002**	**17.6** ***p*** **< 0.001**	**17.9** ***p*** **< 0.001**
4 (*n* = 443,441)	19.5	**26.7** ***p*** **< 0.001**	**23.7** ***p*** **< 0.001**	**24.0** ***p*** **< 0.001**	**22.3** ***p*** **< 0.001**
5 = Most Deprived (*n* = 476,762)	18.9	**28.8** ***p*** **< 0.001**	**31.3** ***p*** **< 0.001**	**37.4** ***p*** **< 0.001**	**32.5** ***p*** **< 0.001**
	**Per cent Distribution by Response Category (Women)**				
	**Heterosexual (Ref.)**	**Lesbian**	**Bisexual**	**Other**	Prefer Not to Say/Missing*
	*n* = 1,021,541	*n* = 6,324	*n* = 4,666	*n* = 8,101	*n* = 177,377
**Age**					
18–24 (*n* = 103,432)	10.3	**13.9** ***p*** **< 0.001**	**25.6** ***p*** **< 0.001**	9.3 *p* = 0.06	**6.5** ***p*** **< 0.001**
25–34 (*n* = 229,680)	17.3	**23.2** ***p*** **< 0.001**	**29.7** ***p*** **< 0.001**	17.3 *p* = 0.99	**12.8** ***p*** **< 0.001**
35–44 (*n* = 325,195)	19.5	**29.3** ***p*** **< 0.001**	18.5 *p* = 0.16	**17.0** ***p*** **< 0.001**	**14.1** ***p*** **< 0.001**
45–54 (*n* = 374,901)	17.8	**21.8** ***p*** **< 0.001**	**11.0** ***p*** **< 0.001**	**13.3** ***p*** **< 0.001**	**13.6** ***p*** **< 0.001**
55–64 (*n* = 427,004)	15.3	**8.0** ***p*** **< 0.0001**	**6.4** ***p*** **< 0.001**	**12.7** ***p*** **< 0.001**	**13.3** ***p*** **< 0.001**
65–74 (*n* = 354,733)	10.5	**2.5** ***p*** **< 0.001**	**3.8** ***p*** **< 0.001**	**12.5** ***p*** **< 0.001**	**14.8** ***p*** **< 0.001**
75–84 (*n* = 221,209)	6.5	**0.7** ***p*** **< 0.001**	**3.3** ***p*** **< 0.001**	**11.6** ***p*** **< 0.001**	**15.8** ***p*** **< 0.001**
85+ (*n* = 65,407)	2.7	**0.7** ***p*** **< 0.001**	**1.7** ***p*** **< 0.001**	**6.3** ***p*** **< 0.001**	**9.1** ***p*** **< 0.001**
**Race/ethnicity**					
White (*n* = 1,821,689)	91.0	**93.9** ***p*** **< 0.001**	**79.3** ***p*** **< 0.001**	**68.2** ***p*** **< 0.001**	**76.5** ***p*** **< 0.001**
Mixed (*n* = 15,872)	0.8	**1.5** ***p*** **< 0.001**	**2.7** ***p*** **< 0.001**	**1.4** ***p*** **< 0.001**	**1.0** ***p*** **< 0.001**
Asian (*n* = 110,904)	3.2	**0.9** ***p*** **< 0.001**	**6.7** ***p*** **< 0.001**	**10.5** ***p*** **< 0.001**	**10.4** ***p*** **< 0.001**
Black (*n* = 56,901)	2.0	**1.2** ***p*** **< 0.001**	**4.7** ***p*** **< 0.001**	**7.3** ***p*** **< 0.001**	**4.9** ***p*** **< 0.001**
Chinese (*n* = 9,859)	0.5	0.3 *p* = 0.08	0.8 *p* = 0.07	**1.7** ***p*** **< 0.001**	**0.9** ***p*** **< 0.001**
Other (*n* = 67,255)	2.4	2.4 *p* = 0.82	**5.8** ***p*** **< 0.001**	**11.0** ***p*** **< 0.001**	**6.3** ***p*** **< 0.001**
**Deprivation Quintiles (IMD)**					
1 = Least Deprived (*n* = 366,364)	21.3	**13.9** ***p*** **< 0.001**	**13.2** ***p*** **< 0.001**	**11.7** ***p*** **< 0.001**	**13.8** ***p*** **< 0.001**
2 (*n* = 402,019)	21.0	**17.5** ***p*** **< 0.001**	**16.1** ***p*** **< 0.001**	**14.2** ***p*** **< 0.001**	**16.0** ***p*** **< 0.001**
3 (*n* = 423,774)	20.5	**18.8** ***p*** **= 0.008**	**18.4** ***p*** **= 0.006**	**16.9** ***p*** **< 0.001**	**18.4** ***p*** **< 0.001**
4 (*n* = 443,441)	19.6	**23.7** ***p*** **< 0.001**	**24.2** ***p*** **< 0.001**	**22.6** ***p*** **< 0.001**	**22.5** ***p*** **< 0.001**
5 = Most Deprived (*n* = 476,762)	17.6	**26.2** ***p*** **< 0.001**	**28.2** ***p*** **< 0.001**	**34.7** ***p*** **< 0.001**	**29.3** ***p*** **< 0.001**

*43,043 men and 53,129 women selected “prefer not to say”, and 67,318 men and 124,248 women did not answer the sexual orientation item

Entries are percentages of the population based on 2,115,335 observations with non-missing gender and weighted with design and non-response weights to improve the representativeness of respondents in terms of age, gender and practice

*P* values are for tests of whether the designated orientation group differs from the heterosexual/straight reference group of the same gender. Cells for which *p* < 0.01 appear in boldface. For age, race/ethnicity and deprivation, overall tests of whether the characteristic differed by sexual orientation were significant at *p* < 0.001

Results for health status adjusted for age, race/ethnicity, and deprivation appear in Table 2. Sexual minorities reported longstanding psychological or emotional conditions at two to three times the adjusted rate of heterosexuals of the same gender (e.g., heterosexual, gay/lesbian, bisexual, other: 5.2 %, 10.9 %, 15.0 %, 10.4 % for men and 6.0 %, 12.3 %, 18.8 % and 9.2 % for women, *p* < 0.001 for all). Sexual minorities were also more likely to report fair/poor general health than corresponding heterosexuals, though the differences were smaller (e.g., 26.4 % of bisexual vs. 19.6 % of heterosexual men, *p* < 0.001 for all). Health disparities by sexual orientation were greater for women than for men, and were consistently larger for bisexuals than for gay and lesbian respondents. Supplementary tables (S2–S3, available online) show that age adjustment was fairly important (as gay, lesbian, and bisexual populations are younger than corresponding heterosexual populations), but there was little additional effect of adjusting for race/ethnicity and deprivation.

**Table 2:** Health Status by Sexual Orientation

	**Percent (95 % confidence interval)**				
**Weighted percentages adjusted for age, race/ethnicity, and deprivation**	**Heterosexual (Ref.) (M:** ***n*** **= 764,291; F:** ***n*** **= 1,021,541)**	**Gay/Lesbian (M:** ***n*** **= 12,346; F:** ***n*** **= 6,324)**	**Bisexual (M:** ***n*** **= 4,161; F:** ***n*** **= 4,666)**	**Other (M:** ***n*** **= 6,167; F:** ***n*** **= 8,101)**	Prefer Not to Say/Missing*(M: ***n*** **= 110,361; F:** ***n*** **= 177,377)**
**Men**					
Fair/Poor General Health Status	19.6 (19.5, 19.7)	**21.9 (20.9, 22.8)** ***p*** **< 0.001**	**26.4 (24.8, 27.9)** ***p*** **< 0.001**	**26.8 (25.2, 28.4)** ***p*** **< 0.001**	**24.4 (24.0, 24.7)** ***p*** **< 0.001**
Longstanding psychological or emotional condition	5.2 (5.2, 5.3)	**10.9 (10.3, 11.6)** ***p*** **< 0.001**	**15.0 (13.5, 16.5)** ***p*** **< 0.001**	**10.4 (9.3, 11.4)** ***p*** **< 0.001**	**7.2 (7.0, 7.5)** ***p*** **< 0.001**
**Women**					
Fair/Poor General Health Status	20.5 (20.4, 20.6)	**24.9 (23.6, 26.2)** ***p*** **< 0.001**	**31.6 (30.0, 33.3)** ***p*** **< 0.001**	**27.1 (26.0, 28.3)** ***p*** **< 0.001**	**24.7 (24.5, 25.0)** ***p*** **< 0.001**
Longstanding psychological or emotional condition	6.0 (5.9, 6.0)	**12.3 (11.4, 13.2)** ***p*** **< 0.001**	**18.8 (17.1, 20.5)** ***p*** **< 0.001**	**9.2 (8.4, 10.1)** ***p*** **< 0.001**	**6.8 (6.6, 7.0)** ***p*** **< 0.001**

*43,043 men and 53,129 women selected “prefer not to say”, and 67,318 men and 124,248 women did not answer the sexual orientation item

Entries are adjusted percentages of the population based on 2,115,335 observations with non-missing gender and weighted with design and non-response weights to improve the representativeness of respondents in terms of age, gender and practice

*P* values are for tests of whether the designated orientation group differs from the heterosexual/straight reference group of the same gender. Cells for which *p* < 0.01 appear in boldface

Table 3 compares healthcare experiences for each sexual minority group with heterosexuals, separately by gender, using case-mix adjusted percentages from Model 1. Negative patient experiences, while rare in general (ranging from 3.6 to 9.3 % across the four items for heterosexual men and women), were about one and a half times more common for lesbian, gay, and bisexual patients than for heterosexual patients (e.g., 5.6 % of gay men vs. 3.6 % of heterosexual/straight men had no confidence/trust in their physician). These differences were statistically significant (*p* < 0.01) for all four patient experience measures for lesbian and gay respondents and for three of four measures for bisexual respondents, with the largest differences for gay men. Men who report “other” orientation show a similar, though weaker, pattern.

**Table 3:** Patient Experience by Sexual Orientation: Adjusted Percentages (Heterosexual as Comparison Group)*

	**Men**†				
	**Heterosexual (Reference)**	**Gay**	**Bisexual**	**Other**	**Prefer Not to Say/Missing**
**Trust and confidence in doctor = Not at all**	3.6 (3.5, 3.7)	**5.6 (5.1, 6.0)** ***p*** **< 0.001**	4.3 (3.7, 4.8) *p* = 0.06	**5.0 (4.5, 5.5)** ***p*** **< 0.001**	**3.9 (3.7, 4.1)** ***p*** **= 0.002**
**Doctor communication: Any item = Poor or very poor**	9.0 (8.9, 9.1)	**13.5 (12.8, 14.2)** ***p*** **< 0.001**	**12.5 (11.2, 13.8)** ***p*** **< 0.001**	**10.4 (9.3, 11.4)** ***p*** **= 0.007**	9.0 (8.8, 9.3) *p* = 0.93
**Nurse communication: Any item = Poor or very poor**	4.2 (4.1, 4.3)	**7.0 (6.4, 7.6)** ***p*** **< 0.001**	**7.3 (6.2, 8.5)** ***p*** **< 0.001**	**6.7 (5.9, 7.5)** ***p*** **< 0.001**	**5.2 (5.0, 5.5)** ***p*** **< 0.001**
**Overall satisfaction = Fairly or very dissatisfied**	3.8 (3.7, 3.8)	**5.9 (5.4, 6.4)** ***p*** **< 0.001**	**4.9 (4.3, 5.5)** ***p*** **= 0.002**	3.8 (3.3, 4.3) *p* = 0.95	3.7 (3.6, 3.9) *p* = 0.73
	**Women‡**				
	**Heterosexual (Reference)**	**Lesbian**	**Bisexual**	**Other**	**Prefer Not to Say/Missing**
**Trust and confidence in doctor = Not at all**	3.9 (3.8, 3.9)	**5.3 (4.7, 5.9)** ***p*** **< 0.001**	**5.3 (4.6, 6.0)** ***p*** **< 0.001**	4.3 (3.8, 4.8) *p* = 0.17	4.0 (3.8, 4.1) *p* = 0.20
**Doctor communication: Any item = Poor or very poor**	9.3 (9.2, 9.4)	**11.7 (10.8, 12.5)** ***p*** **< 0.001**	**12.8 (11.9, 13.7)** ***p*** **< 0.001**	9.2 (8.5, 9.9) *p* = 0.83	9.1 (8.9, 9.4) *p* = 0.23
**Nurse communication: Any item = Poor or very poor**	4.5 (4.5, 4.6)	**7.8 (7.1, 8.4)** ***p*** **< 0.001**	**6.7 (5.9, 7.5)** ***p*** **< 0.001**	5.3 (4.6, 6.1) *p* = 0.02	**5.1 (4.9, 5.3)** ***p*** **< 0.001**
**Overall satisfaction = Fairly or very dissatisfied**	3.9 (3.8, 3.9)	**4.9 (4.3, 5.5)** ***p*** **< 0.001**	4.2 (3.6, 4.8) *p* = 0.31	**2.9 (2.6, 3.2)** ***p*** **< 0.001**	3.7 (3.6, 3.8) *p* = 0.04

*from Model 1, which also includes controls for age, race/ethnicity, self-rated health, and deprivation quintiles

†Sample sizes for men: confidence and trust in doctor *n* = 827,959; doctor communication *n* = 838,022; nurse communication *n* = 699,365; and satisfaction with care *n* = 856,453

**‡**Sample sizes for women: confidence and trust in doctor *n* = 1,127,664; doctor communication *n* = 1,139,857; nurse communication *n* = 1,035,380; and satisfaction with care *n* = 1,161,213

Cells for which *p* < 0.01 appear in boldface

Model 2 estimates within-practice adjusted differences associated with sexual orientation in order to determine whether differences by sexual orientation are due to concentration of sexual minorities in practices with generally low scores. The patterns of statistical significance are similar to those from Model 1 and the adjusted rates of poor experiences are only slightly smaller (see Appendix Table S4, available online), suggesting that differences by sexual orientation are not due to concentration of sexual minorities in practices with low scores.

Secondary analysis using linear mixed effect models of patient experience (see Appendix Table S5, available online) found large and statistically significant variance components for practices in the differences by orientation. These results indicate that in some practices sexual minority patients report experiences no worse than heterosexual patients, while in other practices the differences are considerably larger than the average differences reported in Model 2.

## DISCUSSION

### Principal Findings

Using patient-reported data from a large English national survey, we found that sexual minorities report substantially worse physical and mental health than their same-gendered heterosexual counterparts. The greatest differences are for longstanding psychological or emotional problems. Sexual minorities are two to three times more likely than heterosexual respondents to report these problems, with the differences being greatest for bisexual respondents. Sexual minorities’ experiences of primary health care also tend to be less favorable than those of heterosexual people of the same gender, age, health, and socioeconomic status. Compared with heterosexual patients, sexual minority patients reported negative healthcare experiences about one and a half times as often, with differences generally largest for nurse communication. The magnitude of these differences in reported experiences is generally similar to what has been reported for other sociodemographic factors: larger than those associated with gender and area socioeconomic deprivation, but smaller than differences between some racial/ethnic groups.^22^

Because the differences in patient experience for sexual minority and heterosexual patients were similar within practices and overall, there is little evidence that concentration of sexual minorities in low-performing practices plays a role in explaining their less favorable experience. Disparities vary substantially across practices, with some practices evaluated similarly by sexual minorities and heterosexual patients and others evaluated as much worse by sexual minorities than heterosexual patients.

### Strengths and Weaknesses of Study

Because of its large sample size, with more lesbian, gay, and bisexual respondents than any health survey of which we are aware, the English GPPS provides a unique opportunity to examine the health and healthcare experiences of sexual minorities at a national level and to compare sexual minorities to heterosexual respondents. Our study also has several limitations. Response rates to the survey are low, but in line with other patient experience surveys.^32^,^33^ Furthermore, response rates are only weakly associated with non-response bias in similar probability sample surveys adhering to high process standards of survey methodology.^34^–^38^ Finally, other analyses of data from this survey compared practice means for survey items in practices with higher response rates with those for practices with low response rates, and found only small differences that were almost entirely explained by differences in the demographic composition of practices.^33^ After adjustment for demographic differences (as is done here) patient response rates have virtually no association with practice means. These findings imply that case-mix adjusted GPPS scores are generally free of non-response bias; similar results have been found in non-response analyses of US patient experience data.^39^ Although a patient’s disclosure of a sexual minority identity may be an important mediator of patient experience, we do not have a measure of disclosure status. Because sexual minorities are stigmatized, some people who identify as gay, lesbian, or bisexual may be unwilling to acknowledge their identity in a survey.^40^ The GPPS does not ask about transgender status directly. It is possible that transgender respondents reported as “some other orientation”, “prefer not to say” or may not have reported any orientation. Relatedly, in this study, we were unable to determine the sexual orientation of respondents who described their orientation as “other” or who said they preferred not to answer the question. However, those who described their identity as “other” were more likely to report worse health (both genders) and healthcare experiences (men only) than heterosexuals, patterns similar to those reporting themselves as being sexual minorities. Research on people who reject all the standard labels for sexual orientation (“other”) is sparse. Results of a longitudinal study of sexual minority women show that at any given time, women with “unlabeled” identities are more similar in their sexual attractions and behavior to bisexual women than to lesbian women and suggest that women may adopt different labels over time.^41^

### Comparison of Proportion of Sexual Minorities to Other Studies

The percentages of respondents who self- identity as gay or bisexual (2.2 % of men replying) or lesbian or bisexual (1.1 % of women replying) are similar to the best available survey estimates in the UK (Jolozo et al. 2010), which found that 1.4 % of adults reported either homosexual or bisexual identities.^42^ These estimates are similar to those in a large US survey that found that 2.0 % of adults reported either homosexual or bisexual identities,^3^ but lower than the estimates from a recent smaller survey (approximate *n* = 15,000) in Britain that found that 2.4 % of men and 2.5 % of women reported either homosexual or bisexual identities.^43^ However, the proportion of people who report same-gender sexual activity in surveys is higher than the proportion describing themselves as gay, lesbian, or bisexual, a finding that holds both in the US and in the UK.^44^,^45^

### Possible Explanations and Implications

The concept of minority stress provides one framework in which to interpret our findings. The model describes stigma, prejudice and discrimination as producing a hostile and stressful social environment that leads to poor mental health, and eventually, physical health.^46^ Moreover, the hostile environment may carry over into the medical practice, leading to poor healthcare experiences. Fears of discriminatory treatment by a provider may also lead to patients postponing healthcare, which can further impair health. Our data do not allow us to distinguish which of these (or other) factors account for observed disparities.

As in other surveys, the proportions reporting minority sexual orientations were higher in younger adults, possibly reflecting changing cultural views of homosexuality that have made it less difficult for those in younger cohorts to acknowledge a gay identity. A substantially higher proportion of racial/ethnic minority than white patients reported bisexual or “other” orientation. This may reflect different socio-cultural norms about acceptability or disclosure of minority sexual orientation among racial/ethnic minorities. While there is a greater concentration of sexual minorities in more deprived neighborhoods, as measured by the Index of Multiple Deprivation, there is evidence that sexual minorities hold higher status occupations than others within those postcodes.^42^ In the US, the relative incomes of sexual minorities differ by gender^47^; after controlling for age and education, men who are in partnerships with other men have lower average incomes than heterosexually married men, whereas partnered lesbian women earn substantially more on average than heterosexually married women.^48^

Overall, our results support “the need for development of programmes in the care and public health needs of lesbian, gay and bisexual (LGB) populations.”^49^ While all categories of sexual minorities may be subject to social stress due to stigma, bisexual people may experience additional stress from the limited community for bisexual individuals (biphobia in lesbian and gay communities^50^), which may explain their particularly high incidence of psychological and emotional problems.^51^ This suggests the importance of tailoring such programs to the needs of specific groups of sexual minority patients.

### Future Research

The GPPS is the world’s largest national population-based survey that examines the health and healthcare experiences of sexual minority patients in primary care. It reveals clear disparities in health and healthcare experiences compared with those of heterosexual patients. These findings indicate the need to better identify and meet the needs of sexual minorities. Further investigation is needed regarding the greater health disparities for bisexuals compared to gays and lesbians. Similar research in the US and other national populations is needed to measure the needs of this globally vulnerable population, and further research efforts should aim to explore how these needs can be best met. For example, comparing general practices with large and no disparities between sexual minority and heterosexual patients may inform efforts to close the health care gap between these populations. A measure of disclosure of orientation to providers would help to interpret results in future surveys. Physicians need to be aware of the problems faced by sexual minorities in order to tailor care most appropriately to their individual needs.

## Electronic Supplementary Material

Below is the link to the electronic supplementary material.

Table S1 (DOCX 24 kb)

Table S2 (DOCX 25 kb)

Table S3 (DOCX 27 kb)

Table S4 (DOCX 25 kb)

Table S5 (DOCX 25 kb)
