# Supplementary material for: Sexual Minorities in England Have Poorer Health and Worse Health Care Experiences: A National Survey
Source: J Gen Intern Med. 2014 Sep 5;30(1):9–16. doi: 10.1007/s11606-014-2905-y (PMC4284269; doi:10.1007/s11606-014-2905-y)
Supplement: Supplementary file 1 — (DOCX 24 kb) [file 11606_2014_2905_MOESM1_ESM.docx]

**Table S1. GPPS patient experience items used in analyses**

1. **Last time you saw a doctor at your GP surgery or health centre, how good was the doctor at each of the following? Please put a** x **in one box for each row.**

|  | Very good | Good | Neither good nor poor | Poor | Very poor | Doesn’t apply |
| --- | --- | --- | --- | --- | --- | --- |
| Giving you enough time |  |  |  |  |  |  |
| Asking about your symptoms |  |  |  |  |  |  |
| Listening to you |  |  |  |  |  |  |
| Explaining tests and treatments |  |  |  |  |  |  |
| Involving you in decisions about your care |  |  |  |  |  |  |
| Treating you with care and concern |  |  |  |  |  |  |
| Taking your problems seriously |  |  |  |  |  |  |

1. **Did you have confidence and trust in the doctor you saw?**
2. Yes, definitely
3. Yes, to some extent
4. No, not at all
5. Don’t know/can’t say
6. **Last time you saw a practice nurse at your GP surgery or health centre, how good was the practice nurse at each of the following? Please put a** x **in one box for each row.**

|  | Very good | Good | Neither good nor poor | Poor | Very poor | Doesn’t apply |
| --- | --- | --- | --- | --- | --- | --- |
| Giving you enough time |  |  |  |  |  |  |
| Asking about your symptoms |  |  |  |  |  |  |
| Listening to you |  |  |  |  |  |  |
| Explaining tests and treatments |  |  |  |  |  |  |
| Involving you in decisions about your care |  |  |  |  |  |  |
| Treating you with care and concern |  |  |  |  |  |  |
| Taking your problems seriously |  |  |  |  |  |  |

1. **In general, how satisfied are you with the care you get at your GP surgery or health centre?**
2. Very satisfied
3. Fairly satisfied
4. Neither satisfied nor dissatisfied
5. Fairly dissatisfied
6. Very dissatisfied
