# Supplementary material for: Sexual Minorities in England Have Poorer Health and Worse Health Care Experiences: A National Survey
Source: J Gen Intern Med. 2014 Sep 5;30(1):9–16. doi: 10.1007/s11606-014-2905-y (PMC4284269; doi:10.1007/s11606-014-2905-y)
Supplement: Supplementary file 3 — (DOCX 27 kb) [file 11606_2014_2905_MOESM3_ESM.docx]

**Table S3. Health status and longstanding psychological or emotional condition by sexual orientation* (adjusted percentages)**

|  | **Per cent (Confidence Interval)** | | | | |
| --- | --- | --- | --- | --- | --- |
|  | **Heterosexual (Ref.)**  **(M: n=764,291;**  **F: n=1,021,541)** | **Gay / Lesbian**  **(M: n=12,346;**  **F: n=6,324)** | **Bisexual**  **(M: n=4,161;**  **F: n=4,666)** | **Other**  **(M: n=6,167;**  **F: n=8,101)** | **Prefer Not to Say / Missing**†  **(M: n=110,361;**  **F: n=177,377)** |
| **Fair/Poor General Health Status (Men)** | | | | |  |
| Unadjusted estimate | 19.3  (19.2, 19.4) | 19.2  (18.3, 20.0)  p=0.85 | **25.8**  **(24.1, 27.4)**  **p<0.001** | **30.1**  **(28.6, 31.5)**  **p<0.001** | **27.9**  **(27.6, 28.2)**  **p<0.001** |
| Adjusted for age | 19.3  (19.2, 19.4) | **24.0**  **(23.0, 24.9)**  **p<0.001** | **28.6**  **(26.8, 30.4)**  **p<0.001** | **30.0**  **(28.6, 31.5)**  **p<0.001** | **26.4**  **(26.1, 26.7)**  **p<0.001** |
| Adjusted for age, deprivation and race/ethnicity | 19.6  (19.5, 19.7) | **21.9**  **(20.9, 22.8)**  **p<0.001** | **26.4**  **(24.8, 27.9)**  **p<0.001** | **26.8**  **(25.2, 28.4)**  **p<0.001** | **24.4**  **(24.0, 24.7)**  **p<0.001** |
| **Longstanding psychological or emotional condition (Men)** | | | | |  |
| Unadjusted estimate | 5.2  (5.1, 5.3) | **13.4**  **(12.6, 14.2)**  **p<0.001** | **16.1**  **(14.5, 17.7)**  **p<0.001** | **11.1**  **(9.9, 12.3)**  **p<0.001** | **7.0**  **(6.8, 7.2)**  **p<0.001** |
| Adjusted for age | 5.2  (5.1, 5.3) | **12.5**  **(11.7, 13.2)**  **p<0.001** | **15.7**  **(14.0, 17.4)**  **p<0.001** | **11.2**  **(10.0, 12.3)**  **p<0.001** | **7.3**  **(7.1, 7.5)**  **p<0.001** |
| Adjusted for age, deprivation and race/ethnicity | 5.2  (5.2, 5.3) | **10.9**  **(10.3, 11.6)**  **p<0.001** | **15.0**  **(13.5, 16.5)**  **p<0.001** | **10.4**  **(9.3, 11.4)**  **p<0.001** | **7·2**  **(7.0, 7.5)**  **p<0.001** |
| **Fair/Poor General Health Status (Women)** | | | | |  |
| Unadjusted estimate | 19.5  (19.4, 19.6) | 19.4  (18.2, 20.6)  p=0.86 | **25.8**  **(24.2, 27.4)**  **p<0.001** | **33.1**  **(31.8, 34.3)**  **p<0.001** | **33.1**  **(32.8, 33.3)**  **p<0.001** |
| Adjusted for age | 20.2  (20.1, 20.3) | **25.7**  **(24.5, 26.9)**  **p<0.001** | **33.5**  **(31.7, 35.3)**  **p<0.001** | **30.6**  **(29.5, 31.7)**  **p<0.001** | **27.2**  **(27.0, 27.5)**  **p<0.001** |
| Adjusted for age, deprivation and race/ethnicity | 20.5  (20.4, 20.6) | **24.9**  **(23.6, 26.2)**  **p<0.001** | **31.6**  **(30.0, 33.3)**  **p<0.001** | **27.1**  **(26.0, 28.3)**  **p<0.001** | **24.7**  **(24.5, 25.0)**  **p<0.001** |
| **Longstanding psychological or emotional condition (Women)** | | | | |  |
| Unadjusted estimate | 6.1  (6.0, 6.1) | **14.5**  **(13.3, 15.7)**  **p<0.001** | **19.6**  **(18.1, 21.2)**  **p<0.001** | **8.7**  **(7.9, 9.5)**  **p<0.001** | 6.1  (5.9, 6.2)  p=0.75 |
| Adjusted for age | 6.0  (5.9, 6.0) | **13.3**  **(12.0, 14.7)**  **p<0.001** | **19.3**  **(17.6, 21.0)**  **p<0.001** | **9.2**  **(8.4, 10.1)**  **p<0.001** | **6.7**  **(6.5, 6.9)**  **p<0.001** |
| Adjusted for age, deprivation and race/ethnicity | 6.0  (5.9, 6.0) | **12.3**  **(11.4, 13.2)**  **p<0.001** | **18.8**  **(17.1, 20.5)**  **p<0.001** | **9.2**  **(8.4, 10.1)**  **p<0.001** | **6.8**  **(6.6, 7.0)**  **p<0.001** |

*43,043 men and 53,129 women selected “prefer not to say”, and 67,318 men and 124,248 women did not answer the sexual orientation item.

Entries are percentages of the population based on 2,115,335 observations with non-missing gender and weighted with design and non-response weights to improve the representativeness of respondents in terms of age, gender and practice.

P-values are for tests of whether the designated orientation group differs from the heterosexual/straight reference group of the same gender. Cells for which p<0.01 appear in boldface.
