# Supplementary material for: Sexual Minorities in England Have Poorer Health and Worse Health Care Experiences: A National Survey
Source: J Gen Intern Med. 2014 Sep 5;30(1):9–16. doi: 10.1007/s11606-014-2905-y (PMC4284269; doi:10.1007/s11606-014-2905-y)
Supplement: Supplementary file 4 — (DOCX 25 kb) [file 11606_2014_2905_MOESM4_ESM.docx]

**Table S4. Patient experience by sexual orientation: Adjusted percentages (heterosexual as comparison group) from Model 2***

|  | **Men**† | | | | |  | **Women‡** | | | | |
| --- | --- | --- | --- | --- | --- | --- | --- | --- | --- | --- | --- |
|  | **Hetero-sexual (Reference)** | **Gay** | **Bisexual** | **Other** | **Prefer Not to Say / Missing** |  | **Hetero-sexual (Reference)** | **Lesbian** | **Bisexual** | **Other** | **Prefer Not to Say / Missing** |
| **Trust and confidence in doctor = Not at all** | 3.6  (3.6, 3.7) | **5.4**  **(5.0, 5.9)**  **p<0.001** | 4.4  (3.9, 5.0)  p=0.02 | **4.9**  **(4.3, 5.4)**  **p<0.001** | 3.8  (3.6, 4.0)  p=0.11 |  | 3.9  (3.9, 4.0) | **5.5**  **(4.8, 6.1)**  **p<0.001** | **5.5**  **(4.8, 6.2)**  **p<0.001** | 4.1  (3.7, 4.6)  p=0.46 | 3.9  (3.8, 4.1)  p=0.99 |
| **Doctor communication: Any item = Poor or very poor** | 9.1  (9.0, 9.2) | **13.3**  **(12.6, 14.0)**  **p<0.001** | **12.3**  **(11.0, 13.6)**  **p<0.001** | 10.1  (9.1, 11.1)  p=0.03 | 8.8  (8.6, 9.1)  p=0.06 |  | 9.3  (9.2, 9.4) | **11.8**  **(11.0, 12.7)**  **p<0.001** | **12.6**  **(11.7, 13.5)**  **p<0.001** | 8.9  (8.1, 9.6)  p=0.26 | **9.0**  **(8.8, 9.2)**  **p<0.001** |
| **Nurse communication: Any item = Poor or very poor** | 4.2  (4.2, 4.3) | **6.3**  **(5.7, 7.0)**  **p<0.001** | **7.2**  **(6.0, 8.4)**  **p<0.001** | **6.5**  **(5.7, 7.4)**  **p<0.001** | **5.2**  **(5.0, 5.4)**  **p<0.001** |  | 4.5  (4.5, 4.6) | **7.6**  **(6.9, 8.2)**  **p<0.001** | **6.4**  **(5.7, 7.1)**  **p<0.001** | 5.2  (4.5, 6.0)  p=0.05 | **5.1**  **(4.9, 5.3)**  **p<0.001** |
| **Overall satisfaction = Fairly or very dissatisfied** | 3.8  (3.7, 3.9) | **5.6**  **(5.2, 6.0)**  **p<0.001** | **4.9**  **(4.3, 5.6)**  **p=0.002** | 3.7  (3.2, 4.2)  p=0.79 | 3.6  (3.4, 3.7)  p=0.02 |  | 3.9  (3.8, 3.9) | **5.1**  **(4.5, 5.7)**  **p<0.001** | 4.3  (3.7, 4.9)  p=0.19 | **2.8**  **(2.5, 3.0)**  **p<0.001** | **3.6**  **(3.5, 3.7)**  **p<0.001** |

*Model 2 adds the random practice effect to Model 1. Model 2 also includes controls for age, race/ethnicity, self-rated health, and deprivation quintiles.

†Sample sizes for men: confidence and trust in doctor n=827,959; doctor communication n=838,022; nurse communication n=6\99,365; and satisfaction with care n=856,453.

**‡**Sample sizes for women: confidence and trust in doctor n=1,127,664; doctor communication n=1,139,857; nurse communication n=1,035,380; and satisfaction with care n=1,161,213.

Cells for which p<0.01 appear in boldface.
