# Supplementary material for: Sexual Minorities in England Have Poorer Health and Worse Health Care Experiences: A National Survey
Source: J Gen Intern Med. 2014 Sep 5;30(1):9–16. doi: 10.1007/s11606-014-2905-y (PMC4284269; doi:10.1007/s11606-014-2905-y)
Supplement: Supplementary file 5 — (DOCX 25 kb) [file 11606_2014_2905_MOESM5_ESM.docx]

**Table S5. Patient experience by sexual orientation: coefficients and variance components from linear probability models (heterosexual as comparison group) ***

|  | **Coefficients for Fixed Effects** | | | | **Practice random effects** | | |
| --- | --- | --- | --- | --- | --- | --- | --- |
|  | **Gay / Lesbian** | **Bisexual** | **Other** | **Prefer not to say / Missing** | **Practice variance component**  **(random intercept)** | **Gay / Lesbian x practice variance component (random slope)** | **Bisexual x practice variance component**  **(random slope)** |
| **Men**† | | | | | | | |
| **Trust and confidence in doctor = Not at all** | **0.024 (0.003)**  **p<0.001** | **0.012 (0.004)**  **p=0.005** | **0.016 (0.003)**  **p<0.001** | **0.002 (0.001)**  **p=0.006** | **0.001 (0.000)**  **p<0.001** | **0.022 (0.001)**  **p<0.001** | **0.019 (0.023)**  **p<0.001** |
| **Doctor communication: Any item = Poor or very poor** | **0.055 (0.004)**  **p<0.001** | **0.042 (0.006)**  **p<0.001** | **0.014 (0.004)**  **p<0.001** | -0.003 (0.001)  p=0.01 | **0.002 (0.000)**  **p<0.001** | **0.042 (0.002)**  **p<0.001** | **0.049 (0.003)**  **p<0.001** |
| **Nurse communication: Any item = Poor or very poor** | **0.028 (0.003)**  **p<0.001** | **0.031 (0.005)**  **p<0.001** | **0.029 (0.003)**  **p<0.001** | **0.010 (0.001)**  **p<0.001** | **0.001 (0.000)**  **p<0.001** | **0.024 (0.001)**  **p<0.001** | **0.035 (0.002)**  **p<0.001** |
| **Overall satisfaction = Fairly or very dissatisfied** | **0.023 (0.003)**  **p<0.001** | **0.014 (0.004)**  **p=0.001** | 0.000 (0.003)  p=1.00 | **-0.002 (0.001)**  **p=0.005** | **0.001 (0.000)**  **p<0.001** | **0.019 (0.001)**  **p<0.001** | **0.022 (0.001)**  **p<0.001** |
| **Women‡** | | | | | | | |
| **Trust and confidence in doctor = Not at all** | **0.023 (0.004)**  **p<0.001** | **0.023 (0.005)**  **p<0.001** | 0.003 (0.002)  p=0.17 | 0.000 (0.001)  p=0.69 | **0.001 (0.000)**  **p<0.001** | **0.023 (0.001)**  **p<0.001** | **0.032 (0.001)**  **p<0.001** |
| **Doctor communication: Any item = Poor or very poor** | **0.033 (0.005)**  **p<0.001** | **0.049 (0.006)**  **p<0.001** | -0.004 (0.004)  p=0.28 | **-0.004 (0.001)**  **p<0.001** | **0.002 (0.000)**  **p<0.001** | **0.038 (0.002)**  **p<0.001** | **0.059 (0.003)**  **p<0.001** |
| **Nurse communication: Any item = Poor or very poor** | **0.030 (0.004)**  **p<0.001** | **0.026 (0.005)**  **p<0.001** | **0.008 (0.003)**  **p=0.004** | **0.005 (0.001)**  **p<0.001** | **0.001 (0.000)**  **p<0.001** | **0.027 (0.001)**  **p<0.001** | **0.034 (0.002)**  **p<0.001** |
| **Overall satisfaction = Fairly or very dissatisfied** | **0.018 (0.003)**  **p<0.001** | 0.008 (0.004)  p=0.06 | **-0.013 (0.002)**  **p<0.001** | **-0.003 (0.001)**  **p<0.001** | **0.001 (0.000)**  **p<0.001** | **0.021 (0.001)**  **p<0.001** | **0.021 (0.001)**  **p<0.001** |

*Models also include controls for age, race/ethnicity, self-rated health, and deprivation quintiles.

†Sample sizes for men: confidence and trust in doctor n=827,959; doctor communication n=838,022; nurse communication n=699,365; and satisfaction with care n=856,453.

**‡**Sample sizes for women: confidence and trust in doctor n=1,127,664; doctor communication n=1,139,857; nurse communication n=1,035,380; and satisfaction with care n=1,161,213.

Cells for which p<0.01 appear in boldface.
